# Surgical and Medical Acute Response Training (SMART) Programme: Improving Preparedness Amongst Newly Qualified Doctors to Manage Clinical Emergencies

**DOI:** 10.7759/cureus.101417

**Published:** 2026-01-13

**Authors:** Jade Sangha, Sumirat M Keshwara, Shashi Thej K Narayana

**Affiliations:** 1 Acute Medicine, Warrington and Halton Teaching Hospitals NHS Foundation Trust, Warrington, GBR

**Keywords:** interactive teaching, medical education, medical emergency teams, near-peer teaching, simulation, undergraduate and graduate medical education

## Abstract

Background

Medical school curricula in the United Kingdom (UK) have been reported to inadequately prepare newly qualified doctors to manage medical emergencies. Near-peer practice has been proposed to enhance emergency medical training. This study describes the development of a training session to improve the knowledge and confidence of newly qualified doctors to manage medical emergencies.

Methods

A near-peer, interactive training session was developed to support newly qualified doctors in managing medical emergency scenarios. The Surgical and Medical Acute Response Training (SMART) session was delivered to newly qualified doctors in the induction week prior to their first clinical placements, which included medicine, surgery, psychiatry, and paediatrics. Pre-session and post-session questionnaires were used to assess the self-reported preparedness of newly qualified doctors to manage medical emergencies. Rank biserial correlation was used to estimate effect sizes. The Wilcoxon signed-rank test was used to identify statistically significant differences in ordinal data between the pre-session and post-session questionnaire responses.

Results

Forty newly qualified doctors attended the SMART session, all of whom completed the pre-session questionnaire (response rate = 100%). Thirty-four participants (85%) completed the post-session questionnaire. Thirty participants (75%) reported exposure to simulation training during their undergraduate training. Seven (17.5%) reported that they considered their undergraduate medical training had prepared them to lead medical emergencies. In the pre-session questionnaire, participants indicated the highest confidence in completing airway, breathing, circulation, disability, and exposure (A-E) assessments and situation, background, assessment, and recommendation (SBAR) handovers. The lowest confidence was reported for determining treatment escalation plans. In the post-session questionnaire, a significant and large positive effect was observed in multiple items relating to awareness of medical emergency teams (METs) and roles of allied health professionals, confidence in leading METs, and clinical management of acute scenarios. No statistically significant effect on confidence in completing an A-E assessment was identified.

Conclusions

This study revealed that newly qualified doctors report reduced confidence across several aspects of managing medical emergencies. Participants reported an increase in confidence in managing acute medical emergencies after participating in the near-peer interactive training session. Clinical educators may consider implementing the SMART session to support newly qualified doctors at the beginning of their post-graduate careers.

## Introduction

Medical school graduates in the United Kingdom (UK) typically undertake a two-year foundation programme (UK Foundation Programme (UKFP)), consisting of six four-month rotations across a variety of medical specialities [1]. During this time, foundation doctors often contribute to medical emergency teams (METs) and respond to calls across the hospital for urgent assessment of clinically deteriorating hospital inpatients [2]. Foundation doctors work alongside senior doctors, acute care nurses, and allied health professionals within these teams; however, foundation doctors are often the first to arrive at emergency scenarios and typically have the least clinical experience [3]. This underscores the importance of training new medical graduates to recognise unwell patients, initiate urgent management, and escalate to senior colleagues.

The General Medical Council’s ‘Outcomes for Graduates’ stipulates that the diagnosis and management of acute medical emergencies is a core competency [4]. However, studies have suggested that this outcome is not being adequately achieved [5,6]. A systematic review and a multi-centre qualitative investigation have highlighted that newly qualified doctors in the UK feel underprepared to manage medical emergencies, and inadequate exposure to emergency settings in undergraduate courses is purported to be a primary contributing factor [7,8]. This lack of preparedness is a key factor to address, as suboptimal training has been associated with patient risk [3].

Supervised practice has been suggested to improve the preparedness of newly qualified doctors in managing medical emergencies [9]. Furthermore, near-peer education has been reported to provide a psychologically safe learning environment, in which students and teachers can build a rapport based on shared learning contexts and experiences [10]. Such factors contribute to enhanced concept retention among students and implementation into practice [10-12]. There is a paucity of research assessing the benefits of near-peer medical emergency training for newly qualified doctors.

This study describes a near-peer training session for newly qualified doctors and evaluates its effectiveness for improving graduates’ self-reported preparedness to manage medical emergencies. The primary outcome was the change in self-reported confidence in medical emergency management. Secondary outcomes included changes in awareness and understanding of METs, acute care teams, and treatment escalation planning.

## Materials and methods

Study design, setting, and participants 

This was a single-centre study conducted at Warrington Hospital, UK. The study was registered as a quality improvement project (QIP) with the local trust quality improvement (QI) team in March 2025 (QI registration WHHQIP0449). The project was designed to apply QI methods to enhance local training practices and did not involve activities that would require formal ethical approval according to the Health Research Authority (HRA) guidance. 

All newly qualified doctors beginning their foundation year 1 (FY1) training at the study site were eligible for inclusion in the study. The new FY1 doctors had not yet started their first clinical rotations (which included medicine, surgery, paediatrics, and psychiatry). Pre-existing FY1 or senior grade doctors were not eligible for inclusion in the study and were not invited to participate in the training session.

Forty FY1 doctors attended the training session and were invited to participate in the study. As this was an exploratory analysis, a statistical sample size calculation was not completed. Participation in the training session was voluntary and did not affect participants’ progression with their medical training. 

Surgical and Medical Acute Response Training (SMART) training session

The quality improvement intervention developed was a near-peer training session entitled "SMART: Surgical and Medical Acute Response Training." The session focused on surgical and medical emergencies and did not cover emergencies that may occur in other specialities (e.g., obstetrics, paediatrics). The purpose of the session was to provide a framework for effectively recognising deteriorating patients and managing medical emergency scenarios. 

SMART was delivered in one session lasting two hours during the trust induction in August 2025. SMART was delivered as an interactive lecture by two foundation year 2 (FY2) doctors (both with one year of postgraduate clinical experience). The session materials were developed by an FY2 doctor and an internal medicine training year 1 (IMT1) doctor (with four years of postgraduate clinical experience). 

The objectives of the session were to understand how to approach medical emergencies, to understand the factors considered when determining the escalation status of a patient, to practise complex communication skills, and to practise presenting patients in the situation, background, assessment, and recommendation (SBAR) format and escalating to seniors out of hours. 

**Figure 1:**
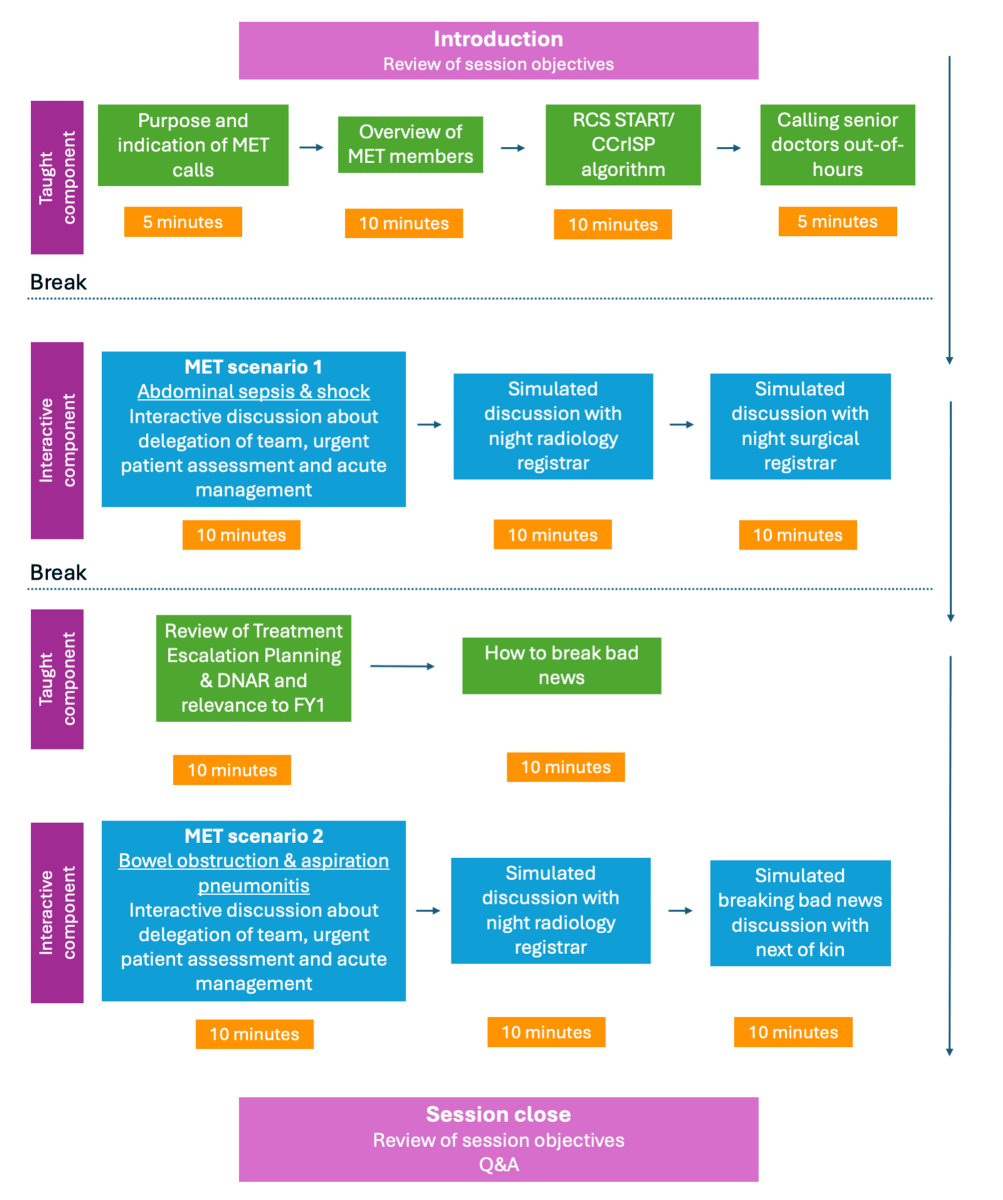
SMART training session workflow SMART: Surgical and Medical Acute Response Training; DNAR: Do Not Attempt Resuscitation; MET: Medical Emergency Team; RCS: Royal College of Surgeons; START: Systematic Training in Acute Illness Recognition and Treatment; CCrISP: Care of the Critically Ill Surgical Patient; FY1: Foundation Year 1; Q&A: Question and Answer The figure is created by the authors.

Figure 1 demonstrates the workflow of the SMART training session. The lecture sequentially covered the session objectives, alternating between taught and interactive components. Participants were taught how to approach medical emergencies in an algorithmic manner, referencing the Royal College of Surgeons’ Systematic Training in Acute Illness Recognition and Treatment (START)/Care of the Critically Ill Surgical Patient (CCrISP) algorithm of managing acutely deteriorating patients [13,14]. Participants were presented with several scenarios in which they had the opportunity to recognise when a patient appeared to be acutely deteriorating. Participants practised complex communication skills, such as breaking bad news and conversations about do not attempt resuscitation (DNAR) decisions and treatment escalation planning (TEP). Participants simulated out-of-hours SBAR handovers to senior members of the surgical and medical teams. 

Data collection and statistical analysis

Prior to the SMART session, participants were invited to voluntarily complete a pre-session questionnaire designed by the authors (Appendix 1). This collected demographic data comprised details such as the medical school where the primary medical qualification was completed and prior exposure to simulation-based training. The questionnaire asked participants to rate their confidence using a five-point Likert scale [15] across multiple domains, including leading medical and surgical emergencies, completing an airway, breathing, circulation, disability, and exposure (A-E) assessment, and identifying the need for emergency surgery. Confidence in functioning as part of a MET was also assessed, encompassing treatment escalation planning, task prioritisation and role allocation, decision-making under pressure, and information gathering in emergency settings. Participants’ awareness of the roles of acute care and medical emergency teams was also evaluated. Following the SMART session, participants completed a post-session questionnaire, which included the same items reassessing these domains.

Statistical analysis was completed using IBM SPSS Statistics Version 30.0 (IBM Corp., Armonk, USA). Ordinal responses from the questionnaire (five-point Likert scale) were treated as approximately interval-level data to enable calculation of mean scores for descriptive comparison across items [16]. The Wilcoxon signed-rank test, a non-parametric test for comparing two paired groups, was used to analyse ordinal data from questionnaire responses. Only participants who completed both the pre-session and post-session questionnaires were included in the analysis assessing the impact of SMART. Participants who had not completed both questionnaires were excluded from this analysis. No data imputation was performed. No correction for multiple testing was performed in this explorative study to reduce the risk of a type 2 error. A p-value of less than 0.05 was considered statistically significant. Effect size (*r*) is reported as a rank biserial correlation and was calculated using the formula *r *= Z/√N, where *Z* is the Wilcoxon test statistic, and *N* is the number of valid paired observations. Effect sizes were interpreted as being negligible (*r *< 0.10), small (0.10 ≤ *r* < 0.30), medium (0.30 ≤ *r* < 0.50), or large (*r* ≥ 0.50) [17].

## Results

Response rate

**Figure 2:**
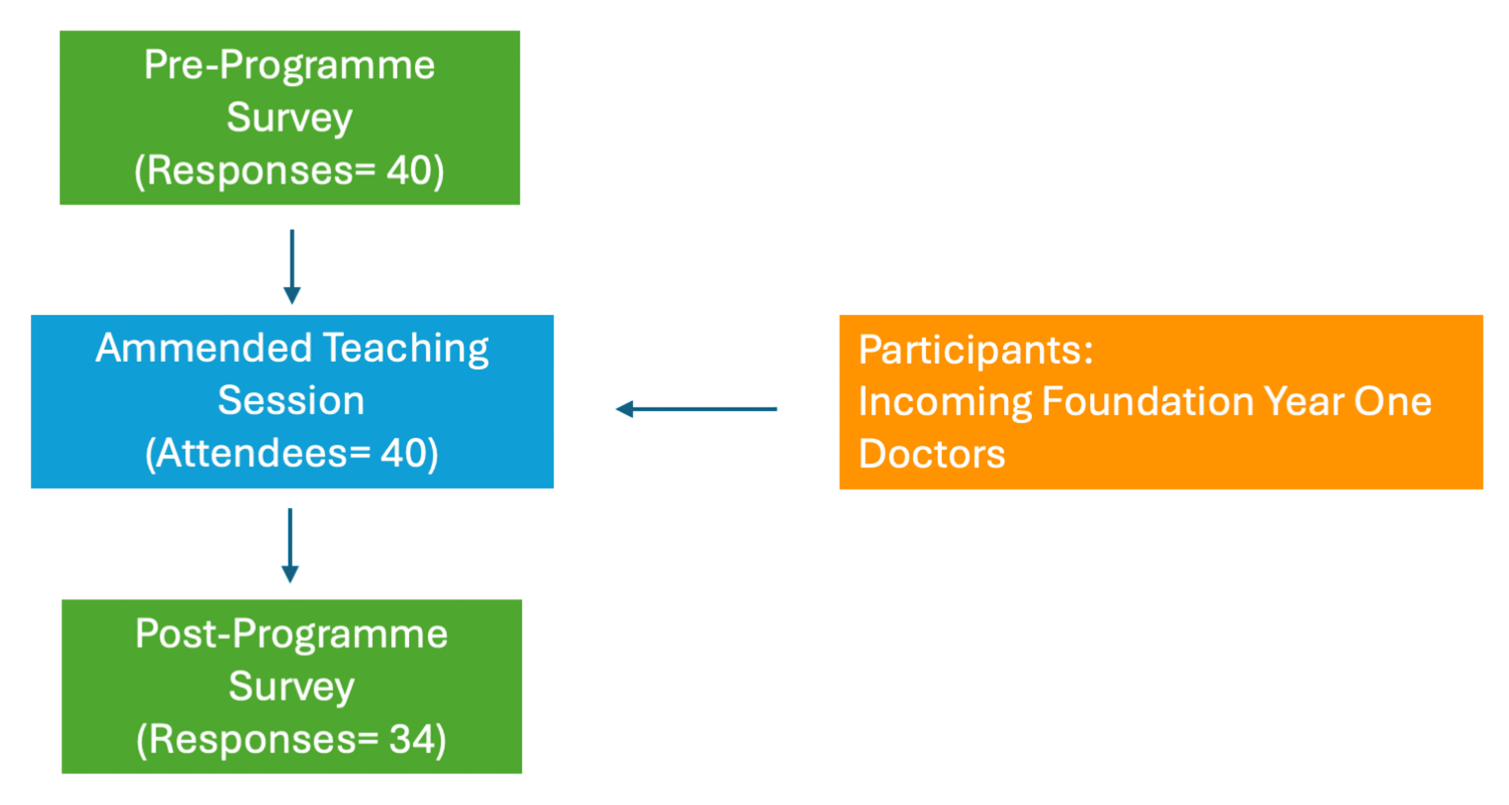
Process flowchart illustrating SMART recruitment SMART: Surgical and Medical Acute Response Training

Figure 2 illustrates participant recruitment. Forty FY1 doctors attended the SMART training session and were invited to participate in the study; all of whom completed the pre-session questionnaire (response rate = 100%). Of those who completed the pre-session questionnaire, 34 FY1 doctors (85%) completed the post-session questionnaire. 

Undergraduate simulation experience

Table 1 demonstrates participants’ self-reported undergraduate simulation experience. Participants had completed their undergraduate medical training at nine different medical schools across the UK. A total of 30 FY1 doctors (75%) reported exposure to simulation training during their undergraduate training. Seven (17.5%) reported that they considered their undergraduate medical training had prepared them to lead medical emergencies.

**Table 1:** Self-reported undergraduate medical training exposure to emergency simulation training MET: Medical Emergency Team

	Response options	Results *N *(%)
Self-reported previous simulation experience	Yes	30 (75)
	No	10 (25)
Self-reported preparedness to lead MET call	Yes	7 (17.5)
	Unsure	10 (25)
	No	23 (57.5)

Preparedness to manage medical emergencies before SMART training

Table 2 demonstrates participant responses for managing medical emergencies before and after the SMART session. In the pre-session questionnaire, participants indicated the highest confidence in completing A-E assessments (mean score 3.8, SD 0.7) and delivering SBAR medical handovers (mean score 3.7, SD 0.7). The lowest confidence was reported for determining treatment escalation plans (mean score 2.4, SD 0.9). 

**Table 2:** Responses to the pre-SMART and post-SMART questionnaire * Statistically significant results (*p* < 0.05) † Large effect size between pre-SMART and post-SMART responses (*r* ≥ 0.5) †† Total of post-SMART responses for item 15 was 33 SMART: Surgical and Medical Acute Response Training; A-E: Airway, Breathing, Circulation, Disability, and Exposure; SBAR: Situation, Background, Assessment, and Recommendation; MET: Medical Emergency Team

	Response options	Before SMART *N* (%) (Total = 40)	After SMART *N *(%) (Total = 34)	*Z* (Number of valid pairs)	r	p
Confidence with completing A-E assessment in emergency setting	Extremely confident	4 (10.0)	3 (8.8)	-1.107 (34)	.190	.268
	Somewhat confident	26 (65.0)	28 (82.4)			
	Neutral	9 (22.5)	2 (5.9)			
	Somewhat not confident	0 (0.0)	1 (2.9)			
	Extremely not confident	1 (2.5)	0 (0.0)			
	Mean (SD)	3.8 (0.7)	4.0 (0.5)			
Confidence with information gathering in emergency setting	Extremely confident	1 (2.5)	1 (2.9)	-3.127 (34)	.536 †	.002*
	Somewhat confident	18 (45.0)	28 (82.4)			
	Neutral	16 (40.0)	5 (14.7)			
	Somewhat not confident	5 (12.5)	0 (0.0)			
	Extremely not confident	0 (0.0)	0 (0.0)			
	Mean (SD)	3.4 (0.7)	3.9 (0.4)			
Confidence with determining treatment escalation plan	Extremely confident	0 (0.0)	1 (2.9)	-3.949 (34)	.677 †	< .001*
	Somewhat confident	6 (15.0)	20 (58.8)			
	Neutral	9 (22.5)	10 (29.4)			
	Somewhat not confident	21 (52.5)	3 (8.8)			
	Extremely not confident	4 (10.0)	0 (0.0)			
	Mean (SD)	2.4 (0.9)	3.6 (0.7)			
Confidence with difficult discussions	Extremely confident	2 (5.0)	1 (2.9)	-2.232 (34)	.383	.026*
	Somewhat confident	17 (42.5)	25 (73.5)			
	Neutral	10 (25.0)	6 (17.6)			
	Somewhat not confident	9 (22.5)	2 (5.9)			
	Extremely not confident	2 (5.0)	0 (0.0)			
	Mean (SD)	3.2 (1.0)	3.7 (0.6)			
Confidence with SBAR handover	Extremely confident	2 (5.0)	4 (11.8)	-2.358 (34)	.404	.018*
	Somewhat confident	25 (62.5)	25 (73.5)			
	Neutral	10 (25.0)	5 (14.7)			
	Somewhat not confident	3 (7.5)	0 (0.0)			
	Extremely not confident	0 (0.0)	0 (0.0)			
	Mean (SD)	3.7 (0.7)	4.0 (0.5)			
Confidence with communication with other members of team	Extremely confident	2 (5.0)	2 (5.9)	-2.744 (34)	.471	.006*
	Somewhat confident	16 (40.0)	25 (73.5)			
	Neutral	17 (42.5)	7 (20.6)			
	Somewhat not confident	5 (12.5)	0 (0.0)			
	Extremely not confident	0 (0.0)	0 (0.0)			
	Mean (SD)	3.4 (0.8)	3.9 (0.5)			
I know the role of MET	Strongly agree	3 (7.5)	9 (26.5)	-3.250 (34)	.557 †	.001*
	Agree	19 (47.5)	21 (61.8)			
	Neutral	10 (25.0)	4 (11.8)			
	Disagree	7 (17.5)	0 (0.0)			
	Strongly disagree	1 (2.5)	0 (0.0)			
	Mean (SD)	3.4 (1.0)	4.2 (0.6)			
I know the role of Acute Care Team	Strongly agree	2 (5.0)	7 (20.6)	-3.980 (34)	.683 †	< .001*
	Agree	11 (27.5)	22 (64.7)			
	Neutral	12 (30.0)	4 (11.8)			
	Disagree	13 (32.5)	0 (0.0)			
	Strongly disagree	2 (5.0)	1 (2.9)			
	Mean (SD)	3.0 (1.0)	4.0 (0.8)			
I understand the indications for a MET call	Strongly agree	2 (5.0)	10 (29.4)	-2.921 (34)	.501 †	.003*
	Agree	22 (55.0)	20 (58.8)			
	Neutral	10 (25.0)	3 (8.8)			
	Disagree	6 (15.0)	1 (2.9)			
	Strongly disagree	0 (0.0)	0 (0.0)			
	Mean (SD)	3.5 (0.8)	4.2 (0.7)			
Confidence with prioritisation of tasks and role management	Extremely confident	0 (0.0)	3 (8.8)	-3.788 (34)	.650 †	< .001*
	Somewhat confident	11 (27.5)	24 (70.6)			
	Neutral	17 (42.5)	5 (14.7)			
	Somewhat not confident	12 (30.0)	2 (5.9)			
	Extremely not confident	0 (0.0)	0 (0.0)			
	Mean (SD)	3.5 (0.8)	3.8 (0.7)			
Confidence with decision-making under pressure	Extremely confident	0 (0.0)	2 (5.9)	-3.573 (34)	.613 †	< .001*
	Somewhat confident	11 (27.5)	21 (61.8)			
	Neutral	17 (42.5)	10 (29.4)			
	Somewhat not confident	12 (30.0)	1 (2.9)			
	Extremely not confident	0 (0.0)	0 (0.0)			
	Mean (SD)	3.0 (0.8)	3.7 (0.6)			
Confidence managing acute respiratory failure	Extremely confident	0 (0.0)	1 (2.9)	-3.241 (34)	.556 †	.001*
	Somewhat confident	7 (17.5)	21 (61.8)			
	Neutral	17 (42.5)	8 (23.5)			
	Somewhat not confident	13 (32.5)	4 (11.8)			
	Extremely not confident	3 (7.5)	0 (0.0)			
	Mean (SD)	2.7 (0.9)	3.6 (0.7)			
Confidence managing cardiac emergencies	Extremely confident	0 (0.0)	1 (2.9)	-3.147 (34)	.540 †	.002*
	Somewhat confident	8 (20.0)	20 (58.8)			
	Neutral	16 (40.0)	8 (23.5)			
	Somewhat not confident	12 (30.0)	4 (11.8)			
	Extremely not confident	4 (10.0)	1 (2.9)			
	Mean (SD)	2.7 (0.9)	3.5 (0.9)			
Confidence managing shock	Extremely confident	0 (0.0)	1 (2.9)	-2.560 (34)	.439	.010*
	Somewhat confident	10 (25.0)	21 (61.8)			
	Neutral	18 (45.0)	8 (23.5)			
	Somewhat not confident	8 (20.0)	2 (5.9)			
	Extremely not confident	4 (10.0)	2 (5.9)			
	Mean (SD)	2.9 (0.9)	3.5 (0.9)			
Confidence managing acute abdomen	Extremely confident	0 (0.0)	2 (6.1)	-3.799 (33 ††)	.661 †	< .001*
	Somewhat confident	6 (15.0)	19 (57.6)			
	Neutral	15 (37.5)	9 (27.3)			
	Somewhat not confident	13 (32.5)	2 (6.1)			
	Extremely not confident	6 (15.0)	1 (3.0)			
	Mean (SD)	2.5 (0.9)	3.6 (0.8)			
Confidence identifying need for emergency surgery	Extremely confident	1 (2.5)	2 (5.9)	-3.503 (34)	.601 †	< .001*
	Somewhat confident	8 (20.0)	23 (67.6)			
	Neutral	16 (40.0)	8 (23.5)			
	Somewhat not confident	12 (30.0)	1 (2.9)			
	Extremely not confident	3 (7.5)	0 (0.0)			
	Mean (SD)	2.8 (0.9)	3.8 (0.6)			

Preparedness to manage medical emergencies after SMART training

Responses from the post-session questionnaire indicated a significant and large positive effect in confidence for determining treatment escalation plans (*r* = .677, *p* < .001), prioritisation of tasks and role management (*r* = .650, *p *< .001), decision making under pressure (*r* = .613, *p* < .001) and information gathering in an emergency setting (*r* = .536, *p* = .002). A significant and large positive effect was identified in participant awareness of the role of acute care (*r* = .683, *p* < .001) and medical emergency teams (*r* = .557, *p* = .001), and indications for MET calls (*r* = .501, *p* = .003). No statistically significant effect in confidence completing an A-E assessment was identified (*r* = .190, *p* = .268).

Regarding clinical scenarios, participants indicated a significant and large positive effect in confidence managing acute abdominal presentations (*r* = .661, *p* < .001), acute respiratory failure (*r* = .556, *p* = .001) and cardiac emergencies (*r* = .540, *p* = .002), and identifying the need for emergency surgery (*r* = .601, *p* < .001).

## Discussion

This study demonstrates that newly qualified doctors in the UK report reduced confidence across several aspects of medical emergency management. Following the implementation of a single-session interactive near-peer training session, study participants reported significantly increased understanding of the roles of acute care and medical emergency teams. Confidence in themes in which participants reported low pre-session confidence, such as treatment escalation planning, identifying the need for emergency surgery, and task prioritisation and role management, underwent large and significant improvement. 

Despite medical schools training students to recognise unwell patients, complete structured assessments, initiate urgent treatment, and communicate bad news, our findings suggest that newly qualified doctors have variable confidence in completing these tasks upon qualification. Undergraduate simulation often focuses on participating in medical emergencies rather than leading, which may reflect the study finding of low pre-session confidence in leading medical emergencies. 

This study may reflect a paucity of exposure to certain aspects of medical emergency management within undergraduate medical curricula. Some aspects of medical emergency management, such as identifying when treatment escalation plans and ceilings of care are appropriate, are infrequently highlighted in undergraduate curricula. This may be because these are typically senior-led tasks. Nevertheless, foundation doctors contribute to the teams that make these decisions and are expected to recognise factors that would influence these decisions. Without undergraduate exposure to how treatment escalation is determined, newly qualified doctors may not appreciate the multifaceted nature of caring for deteriorating patients.

After the SMART session, participants reported an improvement in their understanding of medical emergency and acute care teams. The nomenclature, structure, and implementation of METs vary across hospitals. While graduates may have a broad conceptual understanding of the function of a MET, they may lack insight into how they are implemented in practice, particularly in hospitals where they begin their clinical practice. 

Newly qualified doctors commonly work alongside allied healthcare professionals in the METs. These clinicians typically have several years of acute care experience and can support emergency management of patients through procedures (e.g., arterial blood gases), skills (e.g., advanced cardiopulmonary resuscitation), suggesting management options, and assisting with difficult communication. Furthermore, allied health professionals are often more familiar with local pathways of emergency management than newly qualified doctors (e.g., setting up non-invasive ventilation out of hours) and often work in partnership with intensive care units. Therefore, they can streamline care for unwell patients. It is imperative for newly qualified doctors to recognise their role and work effectively with them to enhance patient care.

Following the SMART session, there was no statistically significant improvement in confidence in completing A-E assessments. Most study participants reported previous exposure to simulation during medical school. Such prior exposure, commonly involving practising A-E assessment and SBAR handover, likely contributed to high pre-session confidence in these areas. Furthermore, these concepts are commonly assessed in undergraduate clinical examinations. The SMART session did not include small group simulation, an approach more conducive to practising A-E assessment and receiving individualised feedback [9,18]. This limitation may account for the lack of significant improvement in this domain. While supervised practice on the wards may not always be feasible due to time, cost, and environmental factors, simulation-based learning can be a suitable alternative, providing a holistic training experience mimicking real clinical practice [10-12]. Participants are encouraged to hone clinical and non-clinical skills, such as communication and teamwork, to address complex clinical scenarios and practice A-E assessment. 

Several private courses offer structured, algorithm-based simulated practice to approach emergency scenarios. However, many do not address the needs of new graduates. START and CCrISP courses aimed at managing critically ill patients, offered by the Royal College of Surgeons of England, are optional, fee-based, and surgically orientated. These factors limit accessibility, and graduates less interested in pursuing surgical careers may not be inclined to attend. The Resuscitation Council's Advanced Life Support (ALS) course is mandatory for graduates to have completed by the end of the second year of foundation training [19]. However, this course largely focuses on the management of cardiac arrests rather than the assessment and escalation of critically unwell patients. No mandatory, nationally standardised, trust-delivered training exists to prepare new graduates for managing deteriorating patients, despite the likelihood of encountering medical emergencies early in their clinical duties.

The SMART session significantly improved confidence in recognising the need for MET calls and escalation of deteriorating patients. This study has demonstrated the benefit of targeted, early education for newly qualified doctors immediately prior to commencing clinical attachments, in which they may be the first or lead responders to deteriorating patients. While previous research has highlighted the lack of self-reported preparedness among medical graduates responding to acute medical emergencies [8], this study presents a reproducible intervention that demonstrably improves participant self-reported confidence in clinical decision making, treatment escalation planning, and leadership during MET calls. 

Challenges and limitations

Several challenges arose during session design and implementation. Initially, the session was designed as a short, interactive lecture introducing key concepts, followed by tailored practical small group simulation exercises. Each small group of two participants would be supported by one or two facilitators, including volunteer FY1 and FY2 doctors, IMT1 trainees, and general practitioner (GP) trainees. However, recruiting sufficient facilitators proved difficult. The scheduled morning session coincided with ward rounds, limiting the availability of potential volunteer senior doctors. Therefore, the session was adapted as described. Furthermore, the recruitment drive was largely delivered by an FY1 and IMT1 doctor. Recruitment support from senior doctors may have assisted with facilitator engagement, including both doctors and acute care clinicians. The provision of more facilitators could increase tailored feedback for participants and the opportunity to simulate more practical A-E assessments.

The SMART session was conducted at a single centre with a relatively small cohort of newly qualified doctors, which may limit generalisability. This study did not assess variables that may affect doctors’ preparedness for managing medical emergencies, as this level of analysis would require a larger number of participants. The outcomes of the study were based on self-reported confidence as identified through the five-point Likert scale. The impact of the session on objective performance or patient outcomes was not assessed. The questionnaire used was not validated. The longer-term impact of the SMART session on self-reported confidence was not assessed. Furthermore, there is a risk of responder bias as a small proportion of participants did not fill out the post-session questionnaire despite taking part in SMART. 

We recommend that the SMART session be trialled with an increased number of participants across different hospitals. The study team welcomes requests for information about how to deliver SMART at other sites.

## Conclusions

This study describes the development of a near-peer emergency training session (SMART) and evaluates its effectiveness in improving newly qualified doctors’ confidence in leading and managing medical emergencies. The study cohort included UK medical graduates beginning their foundation training. Newly qualified doctors reported reduced confidence across several aspects of managing medical emergencies. After the SMART session, participants reported a significant increase in awareness of METs. Confidence in several themes increased, including treatment escalation planning, task prioritisation, and role management. Our study suggests an apparent paucity in simulation-based training in the undergraduate medical curriculum. Medical education providers should be aware that newly qualified doctors may benefit from additional medical emergency simulation training. The SMART session could be modified and delivered nationally to support doctors early in their postgraduate careers.

## Appendices

Appendix 1 

Programme questionnaire given to participants before and after taking part in the Surgical and Medical Acute Response Training (SMART) programme:

Programme Questionnaire 1. What is your participant ID number? This is to avoid duplication of responses

2. Medical school 

3. Have you participated in any Medical Emergency Team (MET) training or simulation exercises? i) Yes ii) No

4. Has your previous medical school training to date prepared you to lead MET calls? i) Yes ii) No iii) Unsure

Confidence in Medical Emergencies 5. Has your previous medical school training to date prepared you to lead MET calls? i) Extremely Confident ii) Somewhat Confident iii) Neutral iv) Somewhat Not Confident v) Extremely Not Confident 

6. How confident are you in gathering critical information in an emergency situation? Critical information includes: presenting complaint, history of presenting complaint, drug history, social and functional history i) Extremely Confident ii) Somewhat Confident iii) Neutral iv) Somewhat Not Confident v) Extremely Not Confident 

7. How confident are you in determining a treatment escalation plan for a patient? (e.g., for Level 3/2/Ward Ceiling of Care) i) Extremely Confident ii) Somewhat Confident iii) Neutral iv) Somewhat Not Confident v) Extremely Not Confident 

8. How confident are you in having difficult discussions about topics such as Do Not Attempt Cardiopulmonary Resuscitation (DNACPR), ceilings of care, and end of life? i) Extremely Confident ii) Somewhat Confident iii) Neutral iv) Somewhat Not Confident v) Extremely Not Confident 

9. How confident are you in performing medical handover by Situation, Background, Assessment, and Recommendation (SBAR) format to a senior colleague? i) Extremely Confident ii) Somewhat Confident iii) Neutral iv) Somewhat Not Confident v) Extremely Not Confident 

10. How confident are you in your ability to manage acute respiratory failure (e.g., hypoxia, respiratory distress) in an emergency situation? i) Extremely Confident ii) Somewhat Confident iii) Neutral iv) Somewhat Not Confident v) Extremely Not Confident 

11. How confident are you in your ability to manage cardiac emergencies (e.g., acute coronary syndrome, fast atrial fibrillation) during a medical emergency? i) Extremely Confident ii) Somewhat Confident iii) Neutral iv) Somewhat Not Confident v) Extremely Not Confident 

12. How confident are you in your ability to manage shock (e.g., hypovolemic, septic, or cardiogenic shock) during a medical emergency? i) Extremely Confident ii) Somewhat Confident iii) Neutral iv) Somewhat Not Confident v) Extremely Not Confident 

Confidence In Surgical Emergencies 13. How confident are you in your ability to handle emergencies involving the acute abdomen? (e.g., bowel obstruction, pancreatitis) during a surgical emergency? i) Extremely Confident ii) Somewhat Confident iii) Neutral iv) Somewhat Not Confident v) Extremely Not Confident 

14. How confident are you in identifying the need for surgical intervention in an emergency situation? i) Extremely Confident ii) Somewhat Confident iii) Neutral iv) Somewhat Not Confident v) Extremely Not Confident 

Confidence in Working as Part of the Emergency Team 15. How confident are you in your ability to effectively communicate with other members of the MET (e.g., nurses, senior doctors, and other healthcare professionals) during an emergency? i) Extremely Confident ii) Somewhat Confident iii) Neutral iv) Somewhat Not Confident v) Extremely Not Confident 

16. I know what the role of the MET is: i) Strongly Agree ii) Agree iii) Neutral iv) Disagree v) Strongly Disagree 

17. I know what the role of the Acute Care Team nurse is: i) Strongly Agree ii) Agree iii) Neutral iv) Disagree v) Strongly Disagree 

18. I understand the reasons why a MET call may be placed: i) Strongly Agree ii) Agree iii) Neutral iv) Disagree v) Strongly Disagree 

19. How confident are you in your ability to prioritise tasks and manage your role in a fast-paced emergency setting? i) Extremely Confident ii) Somewhat Confident iii) Neutral iv) Somewhat Not Confident v) Extremely Not Confident 

20. How confident are you in your ability to make decisions under pressure during a medical or surgical emergency? i) Extremely Confident ii) Somewhat Confident iii) Neutral iv) Somewhat Not Confident v) Extremely Not Confident 
